# Ionizing radiation modulates human macrophages towards a pro-inflammatory phenotype preserving their pro-invasive and pro-angiogenic capacities

**DOI:** 10.1038/srep18765

**Published:** 2016-01-06

**Authors:** Ana Teresa Pinto, Marta Laranjeiro Pinto, Ana Patrícia Cardoso, Cátia Monteiro, Marta Teixeira Pinto, André Filipe Maia, Patrícia Castro, Rita Figueira, Armanda Monteiro, Margarida Marques, Marc Mareel, Susana Gomes dos Santos, Raquel Seruca, Mário Adolfo Barbosa, Sónia Rocha, Maria José Oliveira

**Affiliations:** 1 I3S-Instituto de Investigação e Inovação em Saúde, Universidade do Porto, Porto, 4200-135, Portugal; 2 INEB-Institute of Biomedical Engineering, University of Porto, Porto, 4200-465, Portugal; 3 FEUP-Faculty of Engineering, University of Porto, Porto, 4200-465, Portugal; 4 ICBAS-Institute of Biomedical Sciences Abel Salazar, University of Porto, Porto, 4050-313, Portugal; 5 IPATIMUP-Institute of Molecular Pathology and Immunology, University of Porto, Porto, 4200-465, Portugal; 6 IBMC-Institute for Molecular and Cell Biology, University of Porto, Porto, 4200-465, Portugal; 7 Radiotherapy Service, Centro Hospitalar S. João, EPE, Porto, 4200–319, Portugal; 8 Department of Radiation Oncology and Experimental Cancer Research, Ghent University Hospital, Ghent, B-9000, Belgium; 9 Department of Pathology and Oncology, Faculty of Medicine, University of Porto, Porto, 4200–319, Portugal; 10 Centre for Gene Regulation and Expression, College of Life Sciences, University of Dundee, Dundee, DD1 5EH, UK

## Abstract

In order to improve the efficacy of conventional radiotherapy, attention has been paid to immune cells, which not only modulate cancer cell response to therapy but are also highly recruited to tumours after irradiation. Particularly, the effect of ionizing radiation on macrophages, using therapeutically relevant doses, is not well understood. To evaluate how radiotherapy affects macrophage behaviour and macrophage-mediated cancer cell activity, human monocyte derived-macrophages were subjected, for a week, to cumulative ionizing radiation doses, as used during cancer treatment (2 Gy/fraction/day). Irradiated macrophages remained viable and metabolically active, despite DNA damage. NF-kappaB transcription activation and increased Bcl-xL expression evidenced the promotion of pro-survival activity. A significant increase of pro-inflammatory macrophage markers *CD80*, CD86 and HLA-DR, but not *CCR7*, *TNF* and *IL1B* was observed after 10 Gy cumulative doses, while anti-inflammatory markers *CD163*, *MRC1*, *VCAN* and IL-10 expression decreased, suggesting the modulation towards a more pro-inflammatory phenotype. Moreover, ionizing radiation induced macrophage morphological alterations and increased their phagocytic rate, without affecting matrix metalloproteases (MMP)2 and MMP9 activity. Importantly, irradiated macrophages promoted cancer cell-invasion and cancer cell-induced angiogenesis. Our work highlights macrophage ability to sustain cancer cell activities as a major concern that needs to be addressed to improve radiotherapy efficacy.

Radiation therapy is a widely used and highly cost effective cancer treatment modality^1^,^2^. The biological principle of its application relies mainly on the direct effect on cancer cells, as they usually exhibit higher proliferative rates and impairment in DNA repair mechanisms, when compared to host cells^3^. Although physics and technological evolution in the field of radiotherapy, involving new imaging modalities and more advanced software/equipment, have largely contributed to improve local control, it is necessary to improve radiotherapy targeting, control disease progression and predict treatment outcome^4^,^5^. To achieve the desired effectiveness, new therapeutic strategies, administered concomitantly, before or after radiotherapy, are required^6^. The key may rely on a better understanding of the effect of ionizing radiation on tumour stromal cells, as they are crucial for disease progression and treatment outcome, and are also irradiated^7^,^8^,^9^,^10^. Ionizing radiation-induced cancer cell death releases death-signals, which lead to the recruitment of more immune cells, including monocytes which differentiate into macrophages at the injured region^11^. Within the different immune cells present at the tumour microenvironment, macrophages are particularly relevant, as they constitute, in many tumours, the major inflammatory stromal component and are also known as obligate partners for cancer cell migration, invasion and metastasis^12^,^13^. Due to their sophisticated phagocytic ability, macrophages perform a crucial role in clearing dying cells, contributing to the induction of tolerance, or stimulation of adaptive antitumour immunity^11^,^14^.

Nevertheless, it is not well understood how ionizing radiation, namely clinically relevant doses, directly affects macrophage behaviour as well as macrophage regulation of cancer progression. Till now a clear limitation on determining the clinical effect of ionizing radiation on elements of the tumour microenvironment are the model systems analysed and the dose of radiation used. Ionizing radiation for cancer treatment is usually delivered in a multi-fractionated regimen, with daily doses of typically 2 Gy (5×/week), often delivered during several weeks^15^. However, the majority of the studies aiming to reveal the effect of ionizing radiation on macrophages are performed in mouse models and make use of single, low (<0.1 Gy) or moderate (0.1 Gy–1 Gy) doses^16^,^17^,^18^,^19^,^20^, thus not relevant in a human clinical context.

The present work aims to reveal the effect of fractionated ionizing radiation on human macrophages, mimicking a week of cancer patients’ treatment. In order to achieve this, human monocyte-derived macrophages were differentiated in the presence of M-CSF, a factor involved in the recruitment of monocytes to the tissues, and exposed to cumulative ionizing radiation doses of 2 Gy each, up to 10 Gy. A plethora of functions in macrophages was then characterized. We demonstrate that irradiated macrophages are viable and metabolically active, activate NF-κB, exhibit a reduced anti-inflammatory profile, increased phagocytosis and unaltered MMP-2 and 9-mediated proteolysis. We also evidenced that irradiated macrophages are still able to promote tumour cell invasion and angiogenesis. Overall, our work adds novelty to the current literature and reinforces the idea of targeting macrophage differentiation and/or their molecular targets as a complementary strategy to improve radiotherapy efficacy.

## Results

### Irradiated macrophages are viable and metabolically active, despite DNA damage

**Figure 1:**
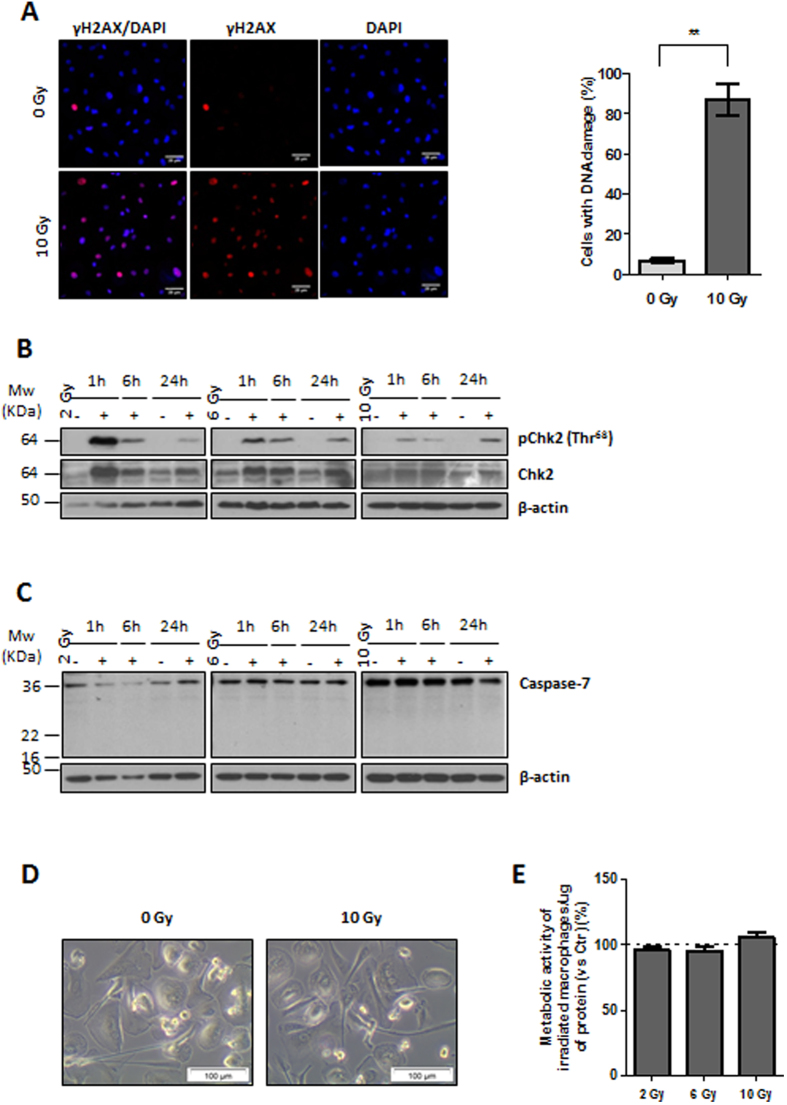
Irradiated human monocyte-derived macrophages are viable and metabolically active, despite DNA damage. (**A**) Radiation-induced macrophage DNA damage is demonstrated by immunocytochemistry for Ser139-phosphorylated H2AX (γH2AX) (red), while nuclei were counterstained with DAPI (blue). Scale bar represents 20 μm. Graph indicates the percentage of macrophages (*n* = 3 and 400 cells/donor counted) exhibiting γH2AX foci. Data was analysed with paired *t*-test. **P < 0.01. (**B**) Western blot analysis of total and phosphorylated Chk2 (Thr68) expression on non-irradiated (−) or irradiated (2, 6 and 10 Gy) (+) macrophages (*n* = 3), upon 1, 6 and 24 h. (**C**) Western blot analysis of caspase-7 expression on non-irradiated (−) or irradiated (2, 6 and 10 Gy) (+) macrophages (*n* = 3), upon 1, 6 and 24 h. In all Western blots β-actin was used as loading control. (**D**) Brightfield microscopic images of non-irradiated (0 Gy) and irradiated macrophages (10 Gy). Scale bar represents 100 μm. (**E**) Quantification of the metabolic activity of irradiated macrophages (2, 6 or 10 Gy) (*n* = 8), normalized to the activity of non-irradiated ones, and expressed as percentage. One-sample *t*-test was performed.

To confirm DNA damage induced by the fractionated irradiation protocol, 10 Gy irradiated macrophages were fixed and immunostained for phosphorylated H2AX (Ser139) (γH2AX), a sensitive marker of DNA double-strand breaks (DSBs)^21^. Immunofluorescence images and respective quantification indicated that a significantly (*P* < 0.01) higher percentage (87.17 ± 7.99%) of irradiated macrophages presented γH2AX foci, in comparison to non-irradiated ones (6.85 ± 1.03%) (Fig. 1A). Upon ionizing radiation exposure, DNA damage signals are generally sensed and propagated through a kinase cascade, including Ataxia Telangiectasia Mutated Kinase (ATM) and also Checkpoint kinase 2 (Chk2)^22^. Therefore, Chk2 expression and phosphorylation status were evaluated by western blot analysis. Chk2 activation was consistently observed in all donors at 1, 6 and 24 h after cumulative ionizing radiation doses (2, 6 and 10 Gy) (Fig. 1B). This data confirms that the irradiation protocol applied induced macrophage DNA damage, from single to several cumulative doses, and that the triggered signalling response involves Chk2 activation.

Depending on the level of radiation-induced DNA damage, cell death may or not occur^22^. Apoptosis is known as the major cell death modality induced by ionizing radiation in cells from the myeloid lineage, which is composed by progenitor and mature effector myeloid cells, such as macrophages, dendritic cells, erythrocytes and platelets^23^,^24^,^25^. In the last years, a heterogeneous population of immature myeloid cells with immunosuppressive properties, termed myeloid-derived suppressor cells (MDSCs), has been defined^26^. Together with macrophages, this population is frequently recruited into tumours after ionizing radiation exposure and less radiosensitive than other lymphocyte subsets^27^,^28^,^29^,^30^.

Complementary approaches to detect cell apoptosis may include evaluation of several cellular aspects, such as caspase activation, cell morphology and mitochondrial status^31^. If apoptosis was occurring in irradiated macrophages, effector caspases, those responsible for the apoptosis execution phase, such as caspase-7, should be functionally activated, this is proteolytically cleaved^31^. Therefore, caspase-7 expression was evaluated by western blot analysis, using an antibody which detects both the full length caspase-7 (35 kDa) and the large fragment of cleaved caspase-7 (20 kDa) (Fig. 1C). We demonstrated that there was no caspase-7 cleavage in irradiated macrophages. Additionally and focusing on the strongest cumulative dose (10 Gy) (6 h time point) (Supplementary Fig. S1), we confirmed, by using a positive control, that caspase-7 was not being cleaved in irradiated macrophages. At the same time, we also evaluated the cleavage of caspase-3, which is is a critical executioner of apoptosis^31^, and the cleavage of PARP, a substrate of several cell death proteases^32^, including caspase-3 (Supplementary Fig. S1). The antibody which detects the full length caspase-3 (35 kDa) also recognizes the large fragment of cleaved caspase-3 (17 kDa). Positive controls for caspase-3 and -7 activation as well as for PARP cleavage are presented in this western blot panel. As demonstrated by membrane overexposure, no cleavage of caspase-3/–7 nor even of PARP were observed in irradiated macrophages, suggesting that apoptosis was not occurring.

In order to identify apoptosis-related morphological features in macrophages after exposure to cumulative ionizing radiation doses, cells were observed and photographed after each irradiation dose. No apoptotic signs, such as cell shrinkage, pyknosis or loss of membrane integrity^31^, were observed in 10 Gy irradiated and non-irradiated macrophages, suggesting that both populations were similarly viable (Fig. 1D).

To complement previous observations, macrophage mitochondrial function was evaluated 20 h after exposure to 2, 6 and 10 Gy cumulative doses, using the resazurin reduction assay (Fig. 1E). Results evidenced that, independently of the dose, ionizing radiation did not affect macrophage metabolic activity.

Overall, we concluded that irradiated macrophages are viable and metabolically active, and do not activate apoptosis, despite radiation-induced DNA damage.

### Ionizing radiation activates macrophage pro-survival and NF-κB signalling pathway

**Figure 2:**
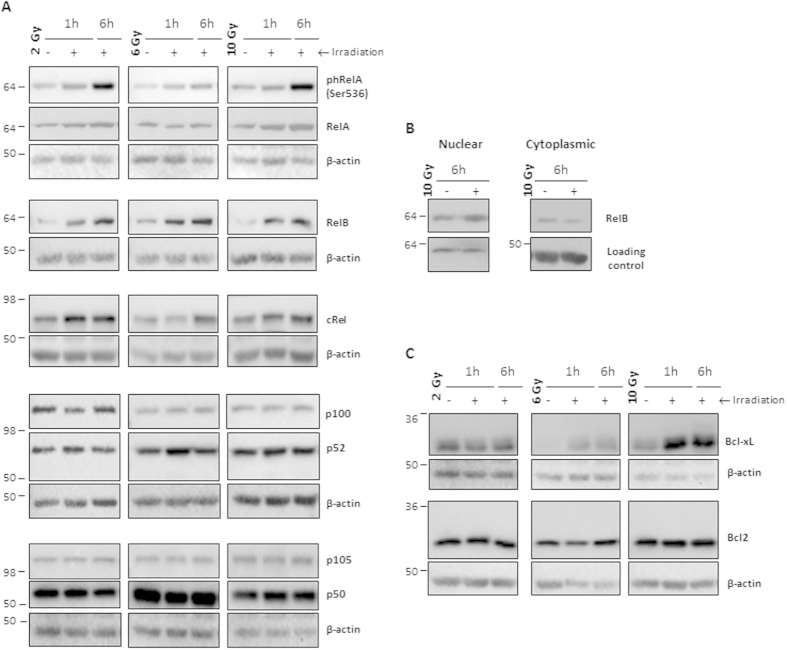
Ionizing radiation induces macrophage NF-κB activation and increases Bcl-xL expression. (**A**) Evaluation of RelA phosphorylation (Ser536) and RelB, cRel, p100/p52 and p105/p50 subunit expression, 1 and 6 h after irradiation (2, 6 and 10 Gy). (**B**) RelB nuclear translocation 6 h after macrophage irradiation (10 Gy). Histone deacetylase 1 (HDAC1) and β-actin were used as loading controls for nuclear and cytoplasmic fractions, respectively. (**C**) Evaluation of Bcl2 and Bcl-xL expression after macrophage irradiation. Western blot images are representative of protein expression/phosphorylation status in distinct donors (at least *n* = 4), evaluated in two independent experiments.

Considering macrophage survival following ionizing radiation exposure, a typical radiation-induced survival pathway mediated by NF-κB^33^, was next investigated. Western blot analysis for the five NF-κB family subunits, RelA, RelB, cRel, p52/p100 and p50/p105 (Fig. 2A) as well as for IκBα (Supplementary Fig. S2), a NF-κB inhibitor, were performed. Densitometry analysis (Supplementary Fig. S3) confirmed that ionizing radiation increased the expression of RelB at every time points in every donors and tend to slightly increase cRel expression (Supplementary Fig. S3A). Additionally, ionizing radiation also tend to slightly increase p100 processing, as suggested by a reduced p52/p100 ratio at 2 and 6 Gy. No major alterations were found in phRel/RelA and p105/p50 levels. Moreover, alterations in RelB suggested that ionizing radiation upregulates macrophage NF-κB, mainly through the non-canonical pathway. As RelB was the most consistently upregulated subunit in response to radiation, we next confirmed RelB nuclear translocation, by evaluating its expression, in nuclear and cytoplasmic extracts, 6 h after 10 Gy cumulative dose (Fig. 2B). In fact, ionizing radiation increased RelB nuclear expression (Supplementary Fig. S3B), suggesting its nuclear translocation and subsequent activation. As NF-κB can induce the expression of anti-apoptotic proteins, such as Bcl2 and Bcl-xL^34^, we evaluated the expression of these targets after macrophage irradiation (2, 6 and 10 Gy) (Fig. 2C). The most relevant alteration was the Bcl-xL increased expression, particularly at 10 Gy (Supplementary Fig. S3C). The increase was visible at 1 h and sustained after 6 h, suggesting induction of macrophage pro-survival activity. Since Bcl-xL maximum expression tends to occur after 10 Gy, all subsequent experiments were performed with macrophages submitted to this cumulative ionizing radiation dose.

### Ionizing radiation induces a reduction in anti-inflammatory macrophage phenotype

**Figure 3:**
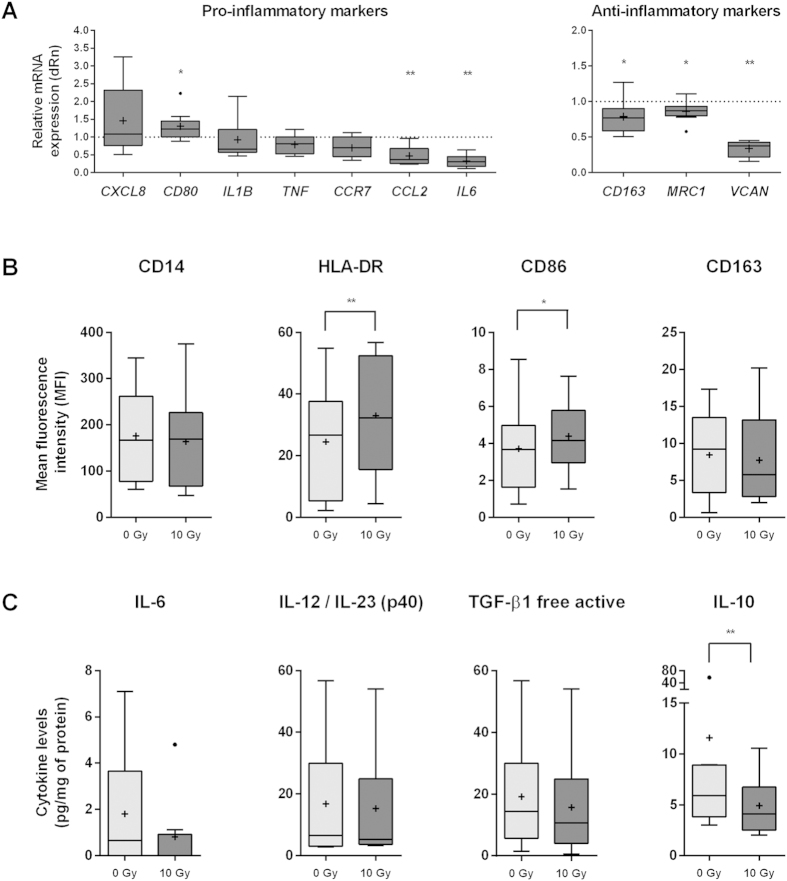
Irradiated macrophages present a reduced anti-inflammatory phenotype. (**A**) mRNA expression profile of pro-(*CXCL8*, *CD80*, *IL1B*, *TNF*, *CCR7*, *CCL2*, *IL6*) and anti-inflammatory (*CD163*, *MRC1*, *VCAN*) macrophage markers, by real-time PCR, 20 h after 10 Gy. Graphs represent mRNA expression of irradiated macrophages compared to non-irradiated ones (dotted line) (at least *n* = 7 per marker). β-actin was used as housekeeping gene. Wilcoxon signed rank test was used to compare the median of each dataset against a hypothetical median value of 1. (**B**) Expression of a monocyte/macrophage lineage (CD14), pro-(HLA-DR and CD86) and anti-inflammatory (CD163) macrophage markers was determined, by flow cytometry, 20 h after irradiation (at least *n* = 6 per marker). Paired *t*-test was used for statistical analysis. (**C**) Levels of pro-(IL-6, IL-12/IL-23(p40)) and anti-inflammatory (TGF-β1 and IL-10) cytokines were determined in macrophage CM (*n* = 9) by ELISA, 20 h after irradiation. Data was normalized to protein concentration. Wilcoxon matched pair test was used for statistical analysis. *P < 0.05, **P < 0.01. Median is represented by the horizontal line inside the box plots, while the average is indicated by a “+”. In IL-6 and IL-10 graphics outliers are also indicated.

To evaluate the effect of ionizing radiation on macrophage polarization profile, we characterized the pattern of expression of pro- or anti-inflammatory cytokines/chemokines and cell surface receptors, by a combination of quantitative real-time PCR, flow cytometry and ELISA. Ionizing radiation induced a significant increase (*P* < 0.05) of *CD80* expression, but not of other pro-inflammatory gene markers. In fact, *IL6* and *CCL2* were significantly downregulated (*P* < 0.01) after macrophage irradiation (Fig. 3A). Furthermore, all anti-inflammatory gene markers tested, *CD163*, *MRC1* and *VCAN* were significantly downregulated (*P* < 0.05 or *P* < 0.01) upon irradiation (Fig. 3A). To investigate how these changes could translate into cell surface receptor expression, flow cytometry was then performed. Results evidenced that ionizing radiation significantly increased HLA-DR (*P* < 0.01) and CD86 (*P* < 0.05) (macrophage pro-inflammatory markers), and tend to decrease CD163 (macrophage anti-inflammatory marker), but did not alter the expression of the monocyte/macrophage lineage marker CD14 (Fig. 3B). To further complete the polarization profile analysis, levels of pro-(IL-6 and IL-12/IL-23(p40)) and anti-inflammatory (TGF-β1 and IL-10) cytokines were evaluated by ELISA, using conditioned medium (CM) from non-irradiated (0 Gy) and irradiated (10 Gy) macrophages, normalized to protein concentration (Fig. 3C). Interestingly, only IL-10 levels were significantly downregulated (*P* < 0.01) by ionizing radiation. IFN-γ and TNF-α levels were also investigated, but were undetectable for both macrophage populations. Altogether, this data suggests that ionizing radiation directs macrophages towards a reduced anti-inflammatory phenotype, without achieving however a complete classical pro-inflammatory profile.

### Upon exogenous stimulation, irradiated macrophages polarize towards pro- or anti-inflammatory phenotypes

**Figure 4:**
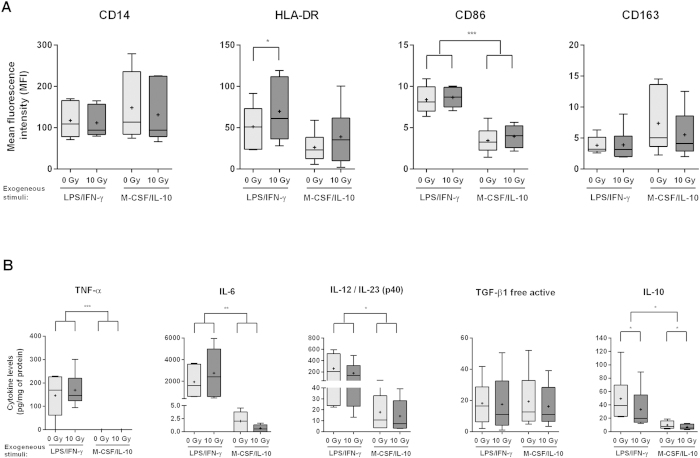
Upon exogenous stimulation, irradiated macrophages polarize towards a pro- or an anti-inflammatory phenotype. Non-irradiated and 10 Gy irradiated macrophages (*n* = 6) were stimulated, for 20 h, with LPS (100 ng/ml) and IFN-γ (20 ng/ml) towards a pro-inflammatory, or with M-CSF (10 ng/ml) and IL-10 (20 ng/ml) towards an anti-inflammatory phenotype. (**A**) Expression of monocyte/macrophage lineage (CD14), pro-(HLA-DR and CD86) and anti-inflammatory (CD163) macrophage markers was determined by flow cytometry. (**B**) Macrophage CM levels of pro-(TNF-α, IL-6, IL-12/IL-23(p40)) and anti-inflammatory (TGF-β1 and IL-10) cytokines were determined by ELISA. Data was normalized to protein concentration. Paired *t*-test and one-way ANOVA were used for statistical analysis. *P < 0.05, **P < 0.01, ***P < 0.001. Median is represented by the horizontal line inside the box plots, while the average is indicated by a “+”.

Unstimulated M-CSF differentiated macrophages typically exhibit a more anti-inflammatory-like phenotype, but they are able to polarize towards a pro- or an anti-inflammatory phenotype, after proper exogenous stimulation^35^,^36^. The functional response to external stimuli, termed plasticity, is one of the major hallmarks of the mononuclear phagocyte system^37^. To assess how ionizing radiation affects macrophage plasticity, we evaluated whether irradiated macrophages were still able to polarize towards a pro- or an anti-inflammatory phenotype, upon proper exogenous stimulation. Therefore, upon 10 Gy cumulative dose exposure, macrophages were stimulated for 20 h with LPS (100 ng/mL) and IFN-γ (20 ng/mL), towards a pro-inflammatory phenotype, or with M-CSF (10 ng/mL) and IL-10 (20 ng/mL), towards an anti-inflammatory one. The expression of pro- and anti-inflammatory cell surface markers and cytokines/chemokines was evaluated by flow cytometry (Fig. 4A) and ELISA (Fig. 4B), respectively. Comparison between exogenously stimulated (LPS/IFN-γ or M-CSF/IL-10) non-irradiated (0 Gy) and irradiated (10 Gy) macrophages indicated that ionizing radiation did not affect macrophage ability to polarize towards a pro- or an anti-inflammatory phenotype, upon proper exogenous stimuli. LPS/IFN-γ-stimulated macrophages (both non-irradiated and irradiated) present higher levels of pro-inflammatory markers such as HLA-DR, CD86, TNF-α, IL-6 and IL-12/IL-23 (p40), being the last four significantly different (from *P* < 0.05 to *P* < 0.001), when compared to M-CSF/IL-10-stimulated macrophages (Fig. 4A,B). On the other side, LPS/IFN-γ-stimulated macrophages tend to present lower levels of the anti-inflammatory marker CD163, comparing to M-CSF/IL-10-stimulated macrophages (Fig. 4A). Our results evidenced that, contrary to expectations, the pro-inflammatory stimuli (LPS/IFN-γ) also induced higher levels of immunosuppressive IL-10 cytokine than the anti-inflammatory ones (M-CSF/IL-10). However, this phenomenon was also previously reported in M-CSF-conditioned dendritic cell precursors, which exhibit a rapid IL-10 release upon LPS stimulation and may therefore participate in the modulation of inflammation and immune response^38^.

Interestingly, irradiated macrophages stimulated with LPS/IFN-γ presented significantly (*P* < 0.05) higher levels of HLA-DR, when compared to their non-irradiated counterparts (Fig. 4A). On the other hand, under stimulation with M-CSF/IL-10, irradiated macrophages tend to exhibit lower CD163 and higher HLA-DR levels, when compared to non-irradiated macrophages (Fig. 4A,B). Also, irradiated macrophages polarized towards a pro- or an anti-inflammatory phenotype presented a significant decrease (*P* < 0.05) in IL-10 levels, when compared to non-irradiated and also exogenous stimulated ones (Fig. 4B). Altogether, these results demonstrate that, upon exogenous stimulation, irradiated macrophages are still able to polarize towards a pro- or an anti-inflammatory phenotype. However, ionizing radiation by itself tends to promote the pro-inflammatory phenotype.

### Ionizing radiation induces macrophage morphological alterations and increases their phagocytic rate, without altering MMP-2 and MMP-9 activities

**Figure 5:**
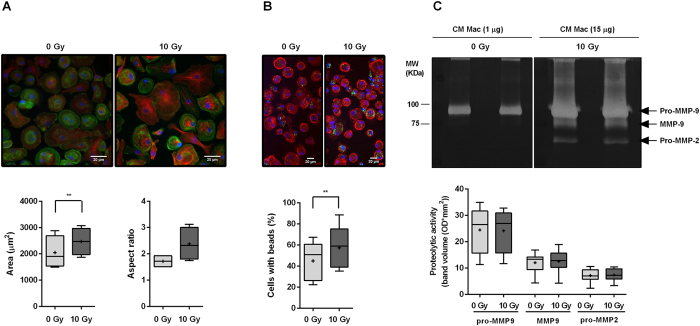
Ionizing radiation increases macrophage area, aspect ratio and phagocytic rate, but does not alter MMP-2 and -9 activities. (**A)** Actin (green) and tubulin (red) stainings of non-irradiated and 10 Gy irradiated macrophages (*n* = 4). Quantification of cell area and aspect ratio was performed using Fiji software. Scale bar indicates 20 μm. (**B**) Phagocytic ability of non-irradiated and 10 Gy-irradiated macrophages (*n* = 5) was determined after 1 h incubation with FITC-labelled (green) *Staphylococcus aureus* particles. F-actin was stained with rhodamine phalloidin (red). The percentage of macrophages able to phagocyte *S.aureus* particles was quantified using Fiji software. Scale bar indicates 20 μm. (**C**) MMP-2 and -9 activity was evaluated by gelatin zymography, using 1 and 15 μg of protein from CM of non-irradiated and 10 Gy-irradiated macrophages (*n* = 10). White bands of proteolytic activity were revealed on a Coomassie Blue-stained gelatin gel. All data was analysed with paired *t*-test. **P < 0.01, ***P < 0.001. Median is represented by the horizontal line inside the box plots, while the average is indicated by a “+”.

In order to characterize morphological changes, macrophage area and aspect ratio were determined 1 h after exposure to 10 Gy (Fig. 5A). Results indicated that irradiated macrophages presented a significantly higher (*P* < 0.05) cell area (2,471 ± 513.3 μm^2^) and tend to increase cell aspect ratio (quotient between major and minor cell axes) (2.38 ± 0.63), when compared to non-irradiated ones (area 2,047 ± 616.3 μm^2^ and aspect ratio 1.72 ± 0.24).

Besides functional and morphological plasticity, other features like phagocytic ability and proteolysis are also characteristic of macrophages^39^. To evaluate the effect of ionizing radiation on macrophage phagocytic ability, non-irradiated and 10 Gy irradiated macrophages were incubated, for 1 h, with FITC-labelled and killed *Staphylococcus (S.) aureus* particles. Results revealed that ionizing radiation significantly increased (*P* < 0.01) the percentage of macrophages able to phagocyte *S. aureus* particles (Fig. 5B).

The impact of ionizing radiation on the activity of MMP-2 and MMP-9, two matrix metalloproteases involved in the promotion of cancer cell invasion and angiogenesis^40^, was determined by gelatin zymography. Using 1 μg of protein from macrophage CM, no differences in pro-MMP-9 proteolytic activity were found, between non-irradiated and 10 Gy irradiated macrophages (Fig. 5C). The same conclusion was extended to MMP-9 and pro-MMP-2 proteolytic bands, when 15 μg of macrophage CM protein were loaded. Our results evidence that ionizing radiation induces macrophage morphologic alterations and increases their phagocytic rate, without affecting macrophage MMP-2 and MMP-9 proteolytic activities.

### Irradiated macrophages promote cancer cell invasion and angiogenesis

**Figure 6:**
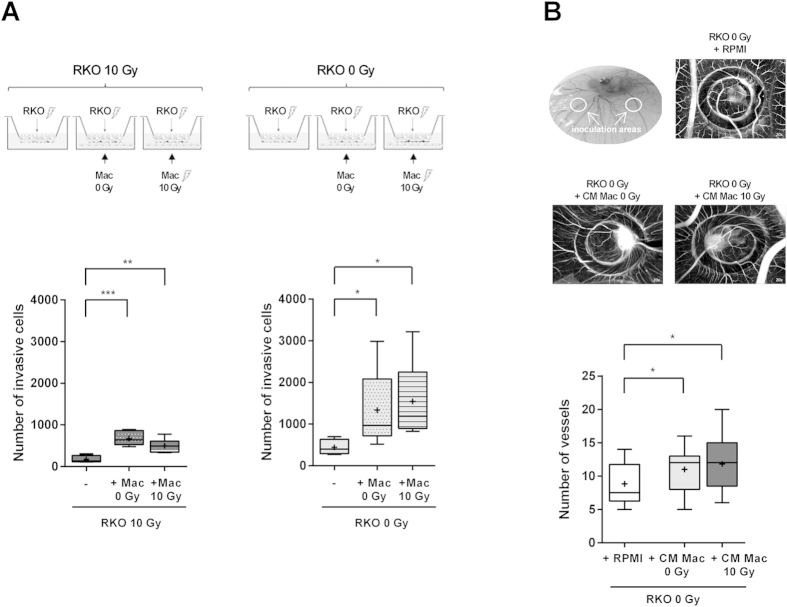
Ionizing radiation does not affect macrophage ability to promote cancer cell invasion and cancer cell-mediated angiogenesis. (**A**) Matrigel invasion assays were established confronting RKO cells (upper compartment) and macrophages (lower compartment) after being separately exposed or not to 10 Gy cumulative ionizing radiation dose. The six possible combinations are represented in the scheme. Invasive cells were counterstained with DAPI and counted on the microscope. (**B**) RKO cells were inoculated, for 72 h, with CM from non-irradiated or 10 Gy irradiated macrophages, in rings (inoculation areas), on top of CAM. The comparison of RKO+ CM Mac 0 Gy versus RKO+CM Mac 10 Gy was evaluated in two rings within the same fertilized egg (*n* = 18), while the condition RKO+RPMI was performed in a single distinct egg (*n* = 16). Analysis of RKO-induced angiogenesis was performed through quantification of the number of new vessels in control and experimental conditions. ANOVA analysis demonstrated a significant difference between groups. *P < 0.05, **P < 0.01, ***P < 0.001. The median is represented by the horizontal line inside the box plots, while the average is indicated by a “+”.

To investigate the effect of ionizing radiation on macrophage pro-invasive cancer cell activity, non-irradiated or 10 Gy irradiated macrophages and irradiated colorectal cancer cells were confronted on Matrigel invasion assays (Fig. 6A). Irradiated as non-irradiated macrophages significantly promoted (*P* < 0.001 or *P* < 0.01) the invasion of irradiated RKO cells. To clarify if macrophage pro-invasive ability was dependent on the presence of irradiated colorectal cancer cells, we used non-irradiated RKO cells. Herein, we verified the same effect previously observed indicating that irradiated macrophages do not lack the ability to promote cancer cell invasion. Moreover, we also observed that irradiation of RKO cells alone significantly decrease (*P* < 0.05) its invasive potential. We could indeed predict that RKO cell invasive potential was going to be highly affected by radiation exposure due to the RKO intrinsic radiosensitivity, previously reported in the literature. An accumulation of about 70% of RKO cells was described in G2/M-phase 16 ± 24 h after 12 Gy irradiation in pH 7.5 medium^41^. RKO cells were also included in a group of radiosensitive cells, upon evaluation of the clonogenic survival of 27 human tumour cell lines after ionizing radiation exposure^42^.

In order to investigate the effect of irradiated macrophages on cancer cell-induced angiogenesis, a chick embryo chorioallantoic membrane (CAM) assay was performed. CM from both non-irradiated and 10 Gy irradiated macrophages significantly promoted (*P* < 0.05) RKO-induced angiogenesis (Fig. 6B). No differences were observed between RKO cells treated with CM derived from non-irradiated and irradiated macrophages. Altogether, Matrigel invasion and CAM-based assays demonstrated that, in the present experimental context, ionizing radiation *per se* was not able to restrain macrophages’ endogenous ability to promote cancer cell invasion and cancer cell–induced angiogenesis, which constitute two main hallmarks of cancer^3^.

## Discussion

The present work aims to understand the effect of ionizing radiation on human macrophages, as they are important components of the tumour microenvironment, and also highly recruited into tumours during radiation therapy^43^. The recurrent use of mouse models and lack of clinically relevant doses in other studies have not allowed a full understanding of this effect^16^,^17^,^18^,^19^,^20^. In the present study, we characterized, for the first time, the response of human primary macrophages to cumulative ionizing radiation doses, using the same fractionated scheme as used during cancer patients’ treatment (2 Gy/fraction/day), up to 10 Gy cumulative dose. As a model, we used M-CSF-cultured macrophages, differentiated from peripheral-blood monocytes, as it is considered the predominant *in vitro* system to study human tissue macrophages^44^. M-CSF is a growth factor involved in the recruitment of monocytes/macrophages to tissues and also in the regulation of macrophage function within tumours^45^. Taking into account that the cellular radiation response is a complex process^46^, several features like DNA damage, NF-κB signalling pathway, polarization profile, plasticity, phagocytosis, proteolysis and the ability to promote cancer cell activities, were evaluated in irradiated human macrophages.

Macrophage resistance to ionizing radiation was first reported in mouse models some decades ago^47^,^48^. Nowadays, it is recognized that human macrophages, similarly to regulatory T cells (Tregs), dendritic cells, Natural Killer (NK) cells and thrombocytes, as well as MDSCs, display a more radiation resistant phenotype than other immune cell populations, such as monocytes^27^,^29^,^30^,^46^. However, most of the studies were performed in mouse macrophages irradiated with X-ray doses, which barely mimic the fractionated scheme used in cancer patients’ treatment. Our results demonstrated that irradiated macrophages exhibited higher DNA damage, confirmed through increased H2AX phosphorylation, than non-irradiated ones. DNA damage induced by ionizing radiation is known to lead to Chk2-specific phosphorylation (Thr68) at sites of DSBs^49^. In agreement, we demonstrated that Chk2 phosphorylation increased along time and according to the exposure doses. Despite DNA damage, irradiated macrophages remain viable and metabolically active, leading to understand which survival pathways were activated. We focused on NF-κB signalling, which is known to induce a pro-survival response in cells exposed to single 2 Gy doses^33^,^50^. Our results demonstrated that RelB expression was consistently increased after macrophage exposure to 2, 6 and 10 Gy cumulative doses. RelB nuclear translocation suggested the involvement of the non-canonical NF-κB pathway in radiation-induced macrophage response. Our data also demonstrated an increased expression of the anti-apoptotic Bcl-xL protein, which also contributes to the promotion of pro-survival activity. Interestingly, a recent study demonstrated that ionizing radiation induces RelB to activate Bcl-xL in cancer cells^51^.

**Figure 7:**
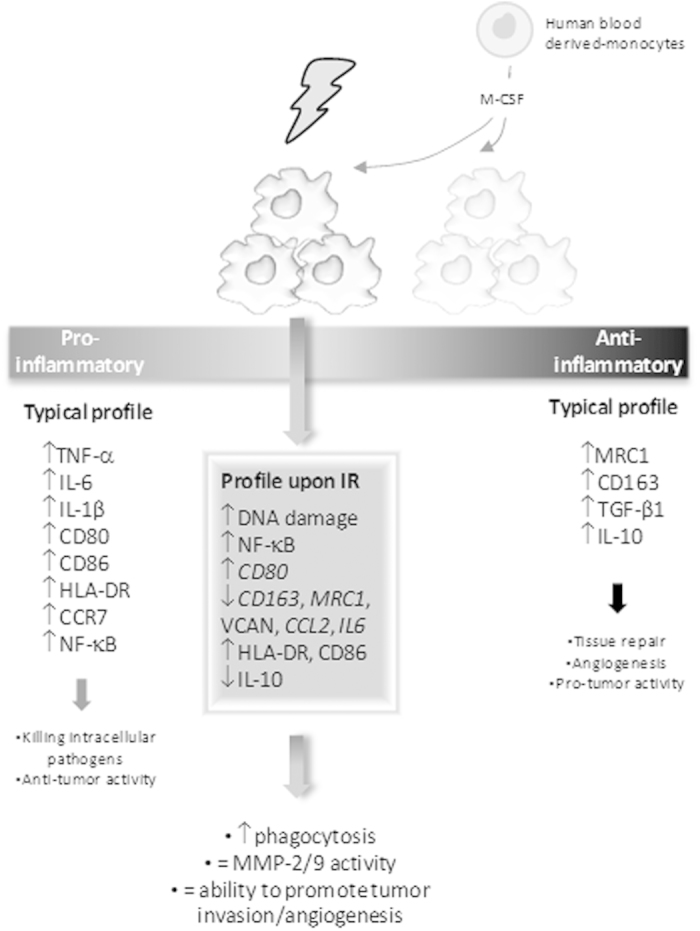
Schematic representation of the effect of ionizing radiation on human blood monocyte-derived macrophages. Two main macrophage functional polarization status are recognized: a pro-inflammatory, responsible for killing intracellular pathogens and antitumour activity, and an anti-inflammatory one, which induces tissue repair, angiogenesis and promotes tumour activity. Pro-inflammatory macrophages produce high levels of TNF-α, IL-6 and IL-1β cytokines and exhibit CD80, CD86, HLA-DR, CCR7 and NF-κB increased expression, while anti-inflammatory ones express CD163, MRC1 and produce high levels of TGF-β1 and IL-10 cytokines. In the present study, we demonstrated that irradiated macrophages exhibit a decrease of anti-inflammatory (*CD163*, *MRC1* and IL-10) and an increase of other pro-inflammatory (*CD80*, CD86, HLA-DR) markers. Although irradiated macrophages are more effective than non-irradiated ones at phagocytosis, a typical feature of pro-inflammatory macrophages, they fail to reach a classical pro-inflammatory phenotype, as they do not produce high levels of TNF-α, IL-6, IL-1β and CCR7. On the other hand, and similarly to their counterparts, irradiated macrophages are able to promote cancer cell invasion and cancer cell-induced angiogenesis. Our data suggests that M-CSF differentiated macrophages, exposed to cumulative ionizing radiation doses up to 10 Gy, exhibit a reduced anti-inflammatory-like phenotype, compared to non-irradiated ones, probably moving towards a pro-inflammatory phenotype. However, although irradiated macrophages exhibit characteristics from both pro- and anti-inflammatory phenotypes, they do not perfectly match to any of these typical profiles, appearing to acquire intermediate characteristics.

NF-κB transcription factors are not only important for cell survival upon irradiation, but are also considered major regulators of inflammation processes and, particularly in macrophages, their activation is required for the anti- to pro-inflammatory phenotype transition^52^,^53^,^54^. Radiation-induced NF-κB alterations in macrophages led to the characterization of macrophage inflammatory status upon irradiation, which data was summarized in the scheme of Fig. 7. In the present study, 10 Gy cumulative ionizing radiation dose significantly decreased both anti-inflammatory gene markers (*CD163*, *MRC1*, *VCAN*) and the immunosuppressive cytokine IL-10, and increased HLA-DR and CD86 expression of M-CSF differentiated macrophages (Fig. 7). Additionally, we also demonstrated that bacterial phagocytosis, a classical feature of pro-inflammatory macrophages, was found to be significantly increased in irradiated macrophages. In fact, mouse macrophages subjected to 8 Gy single dose were also described to slightly enhance phagocytosis of inert latex beads^55^. Together, our data supports the hypothesis that ionizing radiation may drive macrophages towards a pro-inflammatory phenotype. Although irradiated macrophages exhibited increased *CD80* expression, other classical pro-inflammatory markers, like *IL1B*, *TNF* and *IL6* were unaltered, downregulated, or undetectable at the cytokine level. This suggests that irradiated macrophages did not reach a classical pro-inflammatory phenotype, despite the observed reduction of their original anti-inflammatory-like phenotype. The mechanism behind this shift in macrophage phenotype may indeed rely on radiation-induced NF-κB alterations, particularly in RelB subunit as its expression is increased in irradiated macrophages. In fact, very recently, a switch of anti-inflammatory to pro-inflammatory macrophages was found to be directly mediated by RelB induction in M-CSF and TNF-stimulated osteoclast precursors^56^. However, further experiments are still required to confirm RelB involvement in macrophage anti-inflammatory phenotype reduction, suggested to occur after macrophage ionizing radiation exposure.

To simplify the understanding of our hypothesis, we represented the typical pro- and anti-inflammatory macrophage profiles as two extremes of a continuous polarization spectrum (generally represented as a line). However, macrophage polarization has been revealed as a very complex and dynamic system and new representations are emerging as long as new knowledge in this area is coming out. Recently, Ruffell and Coussens summarized the macrophage polarization system as a circle, which does not consider pro- and anti-inflammatory macrophage profiles, but rather emphasize that macrophage functional roles (angiogenesis, cytotoxicity, stimulation, suppression or chemotaxis) (included in an inner circle) are dictated by the integration of multiple stimuli (represented in an outer circle)^57^. Therefore, we may speculate that ionizing radiation exposure could also constitute one of the distinct stimuli capable of polarizing macrophages into a different phenotype, which does not completely corresponds to a pro- or an anti-inflammatory one, but could rather include features from both phenotypes, as we demonstrated in this work, or even from other phenotypes still to be defined. Overall, our study defines, for the first time, a molecular profile for human macrophages subjected to cumulative ionizing radiation doses, emphasizing the important role of fractionated ionizing radiation doses, as used in radiotherapy, to direct macrophages towards a pro-inflammatory phenotype, which is recognized to be tumour cytotoxic^37^. According to the literature, the relation between ionizing radiation exposure and inflammatory response seems to be dependent not only on the cell type analysed and radiation quality, but mainly on the delivered dose^58^. Low doses (maximum of 12 Gy at ≤1.0 Gy/fraction), usually applied in non-malignant disorders or received by normal tissues outside the tumour target volume, induce an anti-inflammatory phenotype, while higher doses (single doses ≥2 Gy, total doses ≥40 Gy) are reported to have a pro-inflammatory effect^59^,^60^. Most of the studies, aiming to reveal the role of irradiation on macrophage inflammatory status, are performed using *in vitro* or *ex vivo* mouse macrophages. Although mouse models have widely contributed to our understanding of ionizing radiation-induced effect on macrophages, it is also well recognized that mouse and human macrophages present many distinct features^61^. Particularly, mouse macrophages are high producers of nitric oxide (NO) and L-citrulline from L-arginine, via inducible nitric oxide synthase (iNOS) activation, while NOS and arginase activities in human macrophages are quite debatable^62^,^63^. These considerations should be taken into account when extrapolating data from one species to another.

We have also demonstrated that irradiated macrophages are as able as their non-irradiated counterparts to promote RKO cancer cell invasion and cancer cell-induced angiogenesis. This is the first report demonstrating that, at least during the first week (5 days) of radiotherapy, human macrophages sustain its ability to promote cancer cell invasion and cancer cell-induced angiogenesis, which is a matter of concern. Nevertheless, we need to be cautious when extrapolating this data to the clinic, as during further neoadjuvant treatment the situation may change. Not only because cell response along treatment time may be different but also because *in vivo* other host cells and several environmental factors may contribute to tumour response to radiotherapy. As our team previously demonstrated, MMP activity is known to be an important factor for macrophage-mediated cancer cell invasion^64^. Therefore, the sustained promotion of cancer cell invasion by irradiated macrophages may be associated with the fact that MMP-2 and MMP-9 activity is not being affected by radiation exposure. Furthermore, we also showed that irradiated macrophages are still able to promote cancer cell-induced angiogenesis.

In summary, our work adds valuable data on characterization of a plethora of functions in human macrophages, subjected to cumulative ionizing radiation doses, as used during cancer patients’ treatment, which were not addressed before. We have characterized important aspects like plasticity, proteolysis, phagocytosis and cancer cell activity promotion. We demonstrated that human macrophages subjected to cumulative doses of ionizing radiation are viable, metabolically active and exhibit increased survival signalling, through NF-κB activation and increased Bcl-xL expression, despite DNA damage. Irradiated macrophages also present a reduced anti-inflammatory profile, increased phagocytosis and unaltered MMP-2 and -9-mediated proteolysis. Pro-inflammatory-like macrophages are known to be cytotoxic and exhibit antitumoural activities and may therefore contribute to the efficacy of local radiotherapy^37^. Our data also demonstrates that irradiation maintains macrophage ability to promote cancer cell invasion and cancer cell-induced angiogenesis. Overall, although radiotherapy mainly induces cancer cell death, other components of the microenvironment, particularly macrophages, are also irradiated and could persist still sustaining the activity of residual radioresistant cancer cells. Furthermore, this knowledge opens new perspectives for macrophage clinical targeting, prior, after or concomitantly to ionizing radiation, as a strategy to improve radiotherapy efficacy.

## Material and methods

### Ethics statement

In the present study, human monocytes were obtained from buffy coats, which are a highly leukocyte-enriched waste-product that results from a whole blood donation, from healthy blood donors. A collaboration protocol between our Institution and Centro Hospitalar São João (CHSJ), where blood donations of Portugal North region are performed, allows the use of these products for investigation purposes. All studies using this human material were approved by CHSJ Ethics Committee for Health (References 259 and 260/11), in agreement with the Helsinki declaration. Informed consent was obtained from all subjects before each blood donation.

### Human monocyte isolation and macrophage differentiation

Human monocytes were isolated as previously described^64^. Following this procedure, over 80% of isolated monocytes were found to be CD14-positive^64^. For monocyte-macrophage differentiation, 1.2 × 10^6^ cells/9.6 cm^2^ (6-well plate) were cultured in complete RPMI1640 medium with GlutaMax (Invitrogen) in the presence of 50 ng/mL of macrophage colony-stimulating factor (M-CSF) (ImmunoTools). Culture medium (1.5 mL/well) was replaced after one week and macrophage differentiation was completed 13 days after monocyte isolation, as at this stage macrophages were shown to provide a higher stimulus for cancer-cell invasion, than with shorter differentiation times^64^.

### Cell culture

RKO (CRL-2577) cells, derived from a human colon carcinoma, were purchased from the American Type Culture Collection (ATCC). Cells were maintained at 37 °C, 5% CO_2_ humidified-atmosphere, in RPMI1640 (L-Glutamine) (Invitrogen) supplemented with 10% FBS (Lonza, Basel, Switzerland), 100 U/mL penicillin and 100 μg/mL streptomycin (Invitrogen).

### Ionizing radiation exposure

Prior irradiation, a dosimetry plan was established (ELEKTA CMS XiO v.4.7.0). Culture plates were submitted to a Computerized Tomography (CT) scan and the volume occupied by two entire plates was defined as the target volume. Two beam fields, one anterior-posterior and other posterior-anterior, were arranged to deliver 2 Gy per fraction to this target volume. Inside the defined volume, the total dose varied from 198 cGy to 202 cGy. As the 4 cGy difference was not significant, the same dose was considered homogenously distributed through plates. To guarantee this uniform dose and to avoid the build-up region of the 18 MV photon beam, 5 water plates were added above, and 5 below the culture plates during irradiation. Medium was renewed before the first irradiation. Both macrophages and RKO cells were then exposed to 1–5 cumulative ionizing radiation doses (2 Gy/fraction/day), for a week (Monday to Friday). Therefore, the maximum cumulative irradiation dose, equivalent to 5 fractions, totalized 10 Gy (Supplementary Fig. S4). Photon beam was produced by a PRIMUS (Siemens, Malvern, PA, USA) linear particle accelerator, used for human radiotherapy sessions, operated at 18 MV at the Radiotherapy Service of CHSJ. To avoid differences between non-irradiated and irradiated cells, caused by medium agitation during transport to/from the Radiotherapy Service, control cells were also transported, but were not radiation-exposed.

### Cell viability

For a proper follow-up of macrophage during irradiation week, cells were carefully observed under a light microscope (Olympus) and daily pictures were taken. To complement this qualitative data, macrophage metabolic activity was determined through resazurin reduction assay, which was considered a sensitive, reproducible and non-destructive assay to measure cell response to irradiation^65^. Briefly, 20 h after irradiation (2, 6 or 10 Gy), macrophages were incubated with resazurin redox dye (0.01 mg/mL) (Sigma-Aldrich) for 3 h at 37 °C and 5% CO_2_. Fluorescence intensity was measured (530 nm Ex/590 nm Em), using the multi-mode microplate reader Synergy MX (BioTek) and values were normalized to protein concentration in the CM, measured with detergent-compatible (DC) protein assay (BioRad). Data from irradiated macrophages was then compared with the respective controls and expressed as percentage.

### Protein extraction and Western Blot

Whole cell protein-extracts were performed 1, 6 and 24 h after irradiation (2, 6 and 10 Gy) (*n* = 4), using lysis buffer supplemented with a cocktail of proteases/phosphatases inhibitors, as previously described^64^. Nuclear/cytoplasmic extracts were performed 6 h after 10 Gy (*n* = 5), using appropriate lysis buffer [10 mM Hepes pH 7.9, 1.5 mM MgCl_2_, 10 mM KCL, 1 mM DTT, 0.1% Igepal, protease/phosphatase inhibitors cocktail]. Cytoplasmic extracts were obtained after centrifugation at 14 000 rpm for 10 min, at 4 °C. For nuclear extracts, pellets were resuspended in another lysis buffer [20 mM Hepes pH 7.9 , 420 mM KCl , 1.5 mM MgCl_2_, 1 mM DTT, 25% Glycerol, protease/phosphatase inhibitors cocktail], rocked for 15 min at 4 °C, centrifuged at 14 000 rpm for 15 min, and sonicated. Following SDS-PAGE, gels were transferred onto polyinylidine difluoride (PVDF) membrane, which were then incubated, for 1 h, with primary antibodies against the following proteins: p105/p50, p100/p52 (Millipore), Bcl-xL (BD Biosciences), phospho-IκBα (Ser32/36) (clone 5A5), IκBα (clone 44D4), phospho-RelA (Ser536) (clone 93H1), Bcl-2 (clone 50E3), phospho-Chk2 (Thr68), Chk2, caspase-3, caspase-7, cleaved PARP (clone D64E10) (Cell Signalling), RelA, RelB, cRel (Santa Cruz Biotechnology). Positive controls for caspase-3 and -7 activation as well as for PARP cleavage were used. Antibody against β-actin (clone 8H10D10) (Cell Signalling) was used to normalize protein expression. Goat anti-rabbit or horse anti-mouse-Horseradish peroxidase (HRP)-conjugated secondary antibodies (Cell Signalling) were used for 1 h, followed by ECL-Detection (Thermo Fisher Scientific). Densitometry analysis of western blot images from Fig. 2 was performed with Quantity One software (BioRad).

### RNA extraction, cDNA preparation and quantitative PCR analysis

Total RNA, from non-irradiated or 10 Gy irradiated macrophages, was extracted using TriPure Isolation Reagent (Roche), according to manufacturer’s instructions. RNA was converted to cDNA using 150 U of SuperScript™ II Reverse Transcriptase, 1× first strand buffer, 10 mM DTT 0.1 M (Invitrogen), 0.5 mM dNTPs 10 mM (Bioron), 8U of rRNasin (Promega) and RNase/DNase free water (Gibco). To evaluate mRNA expression levels of pro- and anti-inflammatory gene markers, quantitative PCR using Brilliant II Sybr green kit (Stratagene/Agilent Technologies) and specific MX3005P 96-well semi-skirted plates, were performed. Samples were analysed on the MX3005P qPCR platform (Stratagene/Agilent). The following primers, from Invitrogen, were used for RT-qPCR: CXCL8, F: 5′-CCAGGAAGAAACCACCGGA-3′, R: 5′-GAAATCAGGAAGGCTGCCAAG-3′; IL1B, F: 5′-GGCAGGGAACCAGCATC-3′, R: 5′-CCGACCACCACTACAGCAA-3′; TNF F: 5′- GGCTGGAGCTGAGAGATA-3′, R: 5′-CAGCCTTGGCCCTTGAAGA-3′. Primer sets for *ACTB* (used as a normalizing gene), *CXCL12* and *CCL2* were obtained from Qiagen, while probes for CD80, CCR7, IL6, CD163, MRC1 and VCAN were from Applied Biosystems.

### Macrophage polarization

After 10 Gy cumulative ionizing radiation exposure, macrophages were stimulated, during 20 h, with 100 ng/mL LPS (Sigma-Aldrich) plus 20 ng/mL IFN-γ (Immunotools) towards a pro-inflammatory phenotype (M1-like), or with 10 ng/mL M-CSF plus 20 ng/mL IL-10 (Immunotools) towards an anti-inflammatory (M2-like) one^44^.

### Flow cytometry

For cell surface receptor expression analysis, non-irradiated and 10 Gy irradiated macrophages, subjected or not to further cytokine-induced polarization, as above detailed, were kept on ice, washed with PBS, gently detached by scraping and resuspended in FACs buffer [PBS, 2% FBS (Lonza), 0.01% sodium azide]. Stainings with anti-human CD14-APC (clone MEM-18), HLA-DR-PE (MEM-12), CD86-FITC (clone BU63) (Immunotools) and CD163-PE (clone GHI/61) (R&D Systems) antibodies were performed in the dark for 30 min. After additional washing steps, macrophages were fixed for 15 min in 4% paraformaldehyde (PFA). Isotype-matched antibodies were used as negative controls, to define background staining. Cells were acquired on a FACS Calibur™ Flow Cytometer (BD Biosciences), using Cell Quest Software (collecting 1 × 10^4^ cells). Analysis was performed with FlowJo software (v7.6.5). Mean fluorescent intensity was calculated by subtracting the respective isotype control intensity.

### Enzyme-linked immunosorbent assay (ELISA)

IL-6, IL-12/IL-23(p40), TNF-α, TGF-β1 free active and IL-10 cytokine levels were determined, according to manufacturer’s instructions (BioLegend), in CM from non-irradiated and 10 Gy irradiated macrophages, subjected or not to further cytokine-induced polarization as above detailed. Briefly, 50 μL of cell culture supernatant were added to a 96-well plate pre-coated with the capture antibody of interest. The soluble proteins bound to the capture antibody were detected using a biotinylated antibody, followed by an avidin-HRP conjugated solution. Finally, the addition of TMB substrate, resulted in a colour change, which intensity was proportional to the amount of antigen captured. Absorbance was then read at 450 and 570 nm. Cytokine levels were determined by plotting values on a standard curve and normalizing them to CM protein concentration.

### Immunocytochemistry

DNA damage and morphology were evaluated, by immunocytochemistry, in non-irradiated and 10 Gy irradiated macrophages (4 × 10^4^). After 1 h, macrophages were fixed with 4% PFA for 20 min and immunocytochemistry procedure was then performed as previously described^64^. Macrophages were incubated with monoclonal antibodies for phosphorylated histone-H2AX (Ser139) (γH2AX) (clone JBW301) (Millipore) or α-tubulin (Sigma-Aldrich), for 1 h, followed by goat-anti mouse AlexaFluor-594-conjugated-secondary antibody (Invitrogen) incubation, for 45 min in the dark. F-actin was stained for 15 min with 0.5 μM Phalloidin-FITC (Sigma-Aldrich), 0.1 M EGTA and 1 M MgSO_4_. Finally, nucleus was stained with 10 μg/mL 4′, 6-diamidino-2-phenylindole (DAPI) solution. Multiwell plate-based screening was performed with a Leica DMI6000 B inverted motorized fluorescence microscope (Leica Microsystems). Microscopic images are represented at 300× magnification.

### Phagocytosis

Ready-made pHrodo green *Staphylococcus aureus* BioParticles Conjugate (1 μm diameter) (Invitrogen) were resuspended in PBS up to 1 mg/mL and gently vortexed and sonicated for homogenous dispersion. Non-irradiated and 10 Gy irradiated macrophages (4 × 10^4^) were then incubated with 1.6 × 10^6^ *S. aureus* particles at 37 °C and 5% CO_2,_ to evaluate phagocytic activity. After 1 h, macrophages were washed in PBS and fixed with 4% PFA for 20 min. For cell identification, F-actin was stained with rhodamine-labeled phalloidin (1:100 dilution) (Invitrogen) for 30 min, after previous permeabilization with 0.2% Triton X-100 and 5% bovine serum albumin (BSA) blocking. Finally, for nuclei visualization, macrophages were incubated with 10 μg/mL DAPI solution for 5 min. Multiwell plate-based screening was performed with IN Cell Analyzer 2000 (GE Healthcare). Microscopic images are represented at 150× magnification. The number of cells able to phagocyte *S. aureus* particles was then determined, using Fiji software^66^.

### Gelatin zymography

Macrophage CM (1 and 15 μg of protein), collected 24 h after 10 Gy cumulative ionizing radiation exposure, were used to evaluate MMP-2 and MMP-9 activities, through gelatin-zymography, as previously described^64^.

### Matrigel invasion assays

To evaluate macrophage-mediated RKO cell invasion, non-irradiated or 10 Gy irradiated RKO cells (5 × 10^4^) were seeded on the upper compartment of Matrigel-coated inserts of 8-μm pore size (BD Biosciences), while non-irradiated or 10 Gy irradiated macrophages (2 × 10^5^), were added on the bottom, for 24 h, as previously described^64^.

### Chick embryo *in vivo* angiogenesis assay

The chick embryo CAM model was used to evaluate RKO-induced angiogenic response in the presence of macrophage CM. Therefore, commercially available fertilized chick (*Gallus gallus*) eggs were horizontally incubated at 37.5 °C, in a humidified atmosphere. On embryonic development day (EDD)3, a square window was opened in the shell after removal of 1.5–2 mL of albumen, to allow detachment of the developing CAM. The window was sealed with a transparent adhesive tape and eggs re-incubated. On EDD10, RKO cells (1 × 10^6^) resuspended in CM from non-irradiated or 10 Gy irradiated macrophages were placed on top of the same CAM, into two independent 3 mm silicone rings, under sterile conditions. For control, RKO cells resuspended in RPMI medium were inoculated in a different egg. Eggs were re-sealed and returned to the incubator for additional 72 h. On EDD13, rings were removed, the CAM was excised from embryos and photographed *ex-ovo* under a stereoscope, using a 20× magnification (Olympus, SZX16 coupled with a DP71 camera). The number of new vessels (<20 μm diameter) growing radially towards the inoculation area was counted in a blind fashion.

### Statistical analysis

All graphs and statistical analysis were performed using GraphPad Prism Software v5 (GraphPad-trial version). Data was analysed for Gaussian distribution using the D’Agostino and Pearson normality test, when *n *≥ 8. To test the hypothesis that irradiated macrophages are different from non-irradiated ones, Wilcoxon matched pairs test was used for non-parametric samples, while *t*-test (either paired *t*-test or one sample *t*-test) was used for parametric data or when *n* < 8. For other comparisons, one-way ANOVA test was performed. Statistical significance was achieved when *P* < 0.05.

## Additional Information

**How to cite this article**: Teresa Pinto, A. *et al*. Ionizing radiation modulates human macrophages towards a pro-inflammatory phenotype preserving their pro-invasive and pro-angiogenic capacities. *Sci. Rep*. **6**, 18765; doi: 10.1038/srep18765 (2016).

## Supplementary Material

Supplementary Information
