# Author Correction: Ionizing radiation modulates human macrophages towards a pro-inflammatory phenotype preserving their pro-invasive and pro-angiogenic capacities

**DOI:** 10.1038/s41598-022-08498-1

**Published:** 2022-03-21

**Authors:** Ana Teresa Pinto, Marta Laranjeiro Pinto, Ana Patrícia Cardoso, Cátia Monteiro, Marta Teixeira Pinto, André Filipe Maia, Patrícia Castro, Rita Figueira, Armanda Monteiro, Margarida Marques, Marc Mareel, Susana Gomes dos Santos, Raquel Seruca, Mário Adolfo Barbosa, Sónia Rocha, Maria José Oliveira

**Affiliations:** 1 I3S-Instituto de Investigação e Inovação em Saúde, Universidade do Porto, 4200-135 Porto, Portugal; 2 INEB-Institute of Biomedical Engineering, University of Porto, 4200-465 Porto, Portugal; 3 FEUP-Faculty of Engineering, University of Porto, 4200-465 Porto, Portugal; 4 ICBAS-Institute of Biomedical Sciences Abel Salazar, University of Porto, 4050-313 Porto, Portugal; 5 IPATIMUP-Institute of Molecular Pathology and Immunology, University of Porto, 4200-465 Porto, Portugal; 6 IBMC-Institute for Molecular and Cell Biology, University of Porto, 4200-465 Porto, Portugal; 7 Radiotherapy Service, Centro Hospitalar S. João, EPE, 4200-319 Porto, Portugal; 8 Department of Radiation Oncology and Experimental Cancer Research, Ghent University Hospital, B-9000 Ghent, Belgium; 9 Department of Pathology and Oncology, Faculty of Medicine, University of Porto, 4200-319 Porto, Portugal; 10 Centre for Gene Regulation and Expression, College of Life Sciences, University of Dundee, Dundee, DD1 5EH UK

Correction to: *Scientific Reports* 10.1038/srep18765, published online 06 January 2016

This Article contains an error in the description of the data presented in Figure 2.

Each blot demonstrating a protein of interest, or of its phosphorylated form, is matched with the expression of β-actin, used as loading control. The majority of the proteins were separated in different gels, apart from proteins p105, p50 and Bcl-xL which were run in the same gel and have the same loading control.

As a result, the Figure 2 legend,

“Ionizing radiation induces macrophage NF-κB activation and increases Bcl-xL expression. (**A**) Evaluation of RelA phosphorylation (Ser536) and RelB, cRel, p100/p52 and p105/p50 subunit expression, 1 and 6 h after irradiation (2, 6 and 10 Gy). (**B**) RelB nuclear translocation 6 h after macrophage irradiation (10 Gy). Histone deacetylase 1 (HDAC1) and β-actin were used as loading controls for nuclear and cytoplasmic fractions, respectively. (**C**) Evaluation of Bcl2 and Bcl-xL expression after macrophage irradiation. Western blot images are representative of protein expression/phosphorylation status in distinct donors (at least *n* = 4), evaluated in two independent experiments.”

should read:

“Ionizing radiation induces macrophage NF-κB activation and increases Bcl-xL expression. (**A**) Evaluation of RelA phosphorylation (Ser536) and RelB, cRel, p100/p52 and p105/p50 subunit expression, 1 and 6 h after irradiation (2, 6 and 10 Gy). (**B**) RelB nuclear translocation 6 h after macrophage irradiation (10 Gy). Histone deacetylase 1 (HDAC1) and β-actin were used as loading controls for nuclear and cytoplasmic fractions, respectively. (**C**) Evaluation of Bcl2 and Bcl-xL expression after macrophage irradiation. Western blot images are representative of protein expression/phosphorylation status in distinct donors (at least *n* = 4), evaluated in two independent experiments. The β-actin loading control of the panels comprised by p105, p50 (2A) and Bcl-xL (2C) is the same, since proteins were separated in the same gel electrophoresis.”

