# Supplementary material for: Ionizing radiation modulates human macrophages towards a pro-inflammatory phenotype preserving their pro-invasive and pro-angiogenic capacities
Source: Sci Rep. 2016 Jan 6;6:18765. doi: 10.1038/srep18765 (PMC4702523; doi:10.1038/srep18765)
Supplement: Supplementary Information [file srep18765-s1.doc]

**Ionizing radiation modulates human macrophages towards a pro-inflammatory phenotype preserving their pro-invasive and pro-angiogenic capacities**

**Ana Teresa Pinto^1,2,3^, Marta Laranjeiro Pinto^1,2,4^, Ana Patrícia Cardoso^1,2,3^, Cátia Monteiro^1,2^, Marta Teixeira Pinto^1,5^, André Filipe Maia^1,6^, Patrícia Castro^1,5^, Rita Figueira^7^, Armanda Monteiro^7^, Margarida Marques^7^, Marc Mareel^8^, Susana Gomes dos Santos^1,2,4^, Raquel Seruca^1,5,9^, Mário Adolfo Barbosa^1,2,4^, Sónia Rocha^10^, and Maria José Oliveira^1,2,9,*^**

^1^I3S-Instituto de Investigação e Inovação em Saúde, Universidade do Porto, Porto, 4200-135, Portugal

^2^INEB-Institute of Biomedical Engineering, University of Porto, Porto, 4150-180, Portugal

^3^FEUP-Faculty of Engineering, University of Porto, Porto, 4200-465, Portugal

^4^ICBAS-Institute of Biomedical Sciences Abel Salazar, University of Porto, Porto, 4050-313, Portugal

^5^IPATIMUP-Institute of Molecular Pathology and Immunology, University of Porto, Porto, 4200-465, Portugal

^6^IBMC-Institute for Molecular and Cell Biology, University of Porto, Porto, 4150-180, Portugal

^7^Radiotherapy Service, Centro Hospitalar S. João, EPE, Porto, 4200–319, Portugal

^8^Department of Radiation Oncology and Experimental Cancer Research, Ghent University Hospital, Ghent, B-9000, Belgium

^9^Department of Pathology and Oncology, Faculty of Medicine, University of Porto, Porto, 4200–319, Portugal

^10^Centre for Gene Regulation and Expression, College of Life Sciences, University of Dundee, Dundee, DD1 5EH, UK

* mariajo@ineb.up.pt

**Supplemental file**


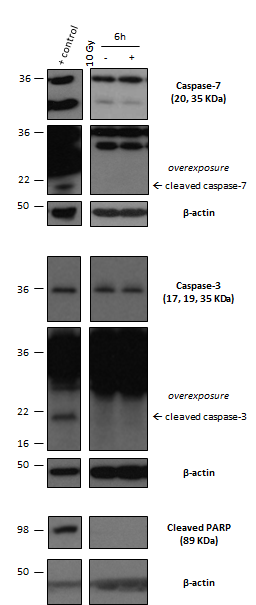


**Supplementary Figure S1.** **Irradiated macrophages do not exhibit caspase-3/-7 nor PARP cleavage.** Expression levels of total caspase-3, caspase-7 and cleaved PARP were evaluated by western blot analysis (*n* = 4) in macrophages 6 h after 10 Gy cumulative dose exposure. β-actin was used as loading control. Positive controls for caspase-3/-7 and PARP cleavage are also present.

**Supplementary Figure S2. Irradiated macrophages do not exhibit phosphorylation of IκBα.** Evaluation of total and phosphorylated IκBα (Ser32/36) levels, by western blot analysis in macrophages 1, 6 and 24 h after 2, 6 and 10 Gy cumulative doses (*n* = 4).

**Supplementary Figure S3. Irradiated macrophages exhibit increase of RelB expression, RelB nuclear translocation and Bcl-xL expression.** Densitometry analysis of western blot images presented in (**A**) Fig. 2A, (**B**) Fig. 2B and (**C**) Fig. 2C. Densitometry analysis was performed with Quantity One software. Statistical analysis was performed with one-sample *t*-test. ** *P* < 0.01


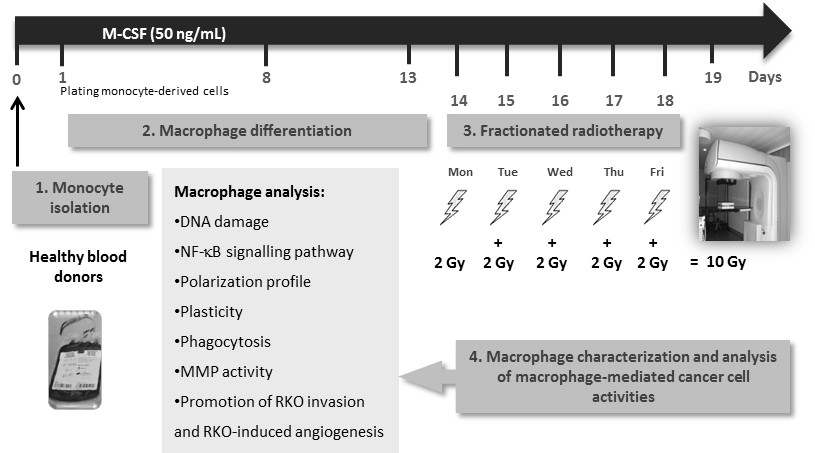


**Supplementary Figure S4. Schematic overview of the methodology used in this work.** Monocytes were isolated from human healthy blood donors’ buffy coats, seeded at day 1, and cultured in the presence of 50 ng/mL macrophage colony-stimulating factor (M-CSF) to allow their differentiation. Upon thirteen days, macrophages and RKO colorectal cancer cells were then X-ray irradiated with daily doses of 2 Gy for 5 days. Upon 10 Gy of cumulative ionizing radiation dose, the direct effect of ionizing radiation on macrophages and on macrophage-mediated RKO cell activities were evaluated, as listed.
